# 2,3-Diphosphoglycerate: the forgotten metabolic regulator of oxygen affinity

**DOI:** 10.1017/S0007114525105345

**Published:** 2025-11-28

**Authors:** Layal S. Jaafar, Christina Mary R. Kourie, Carla A. El-Mallah, Omar Obeid

**Affiliations:** Faculty of Agricultural and Food Sciences, Department of Nutrition and Food Sciences, American University of Beirut, Beirut P.O. Box 11-0236, Lebanon

**Keywords:** 2,3-Diphosphoglycerate, Oxygen affinity, Hb, Erythrocyte, Glycolysis

## Abstract

2,3-Diphosphoglycerate (2,3-DPG), found primarily in red blood cells, plays a key role in regulating hemoglobin’s (Hb) affinity for oxygen. Increased 2,3-DPG levels shift the oxygen dissociation curve to the right, reducing Hb’s oxygen affinity and enhancing oxygen delivery to tissues—particularly important in conditions like anemia and high-altitude adaptation. Despite its physiological significance, research on 2,3-DPG is outdated and limited. This review aims to summarize current knowledge and identify research gaps. Measuring 2,3-DPG is challenging due to its instability and the need for careful sample handling. Chromatography and enzymatic methods are commonly used. Several factors influence 2,3-DPG levels, including diet, physiological state, and disease. Dietary phosphorus, for example, can acutely affect 2,3-DPG levels, though the impact of different meal compositions remains unexplored. Age, pregnancy, and physical activity also modulate 2,3-DPG, yet little is known about its role in infants and children. While changes in 2,3-DPG levels under various pathological conditions have been described, the molecular mechanisms behind these alterations remain poorly understood and warrant further investigation.

2,3-Diphosphoglycerate (2,3-DPG), also known as 2,3-bisphosphoglycerate, a small organic phosphate molecule (MW 266·04 g/mol), was first isolated and discovered in porcine, canine and human erythrocytes and was proposed as *diphospho-l-glyceric acid* in 1925 by Greenwald^(1)^. As the latter was found to be present in a concentration similar to that of Hb (4–5 mmol/l), it was later suggested to be a potent allosteric regulator of Hb by Reinhold and Ruth Benesch in 1967. They reported that intraerythrocytic di- and triphosphates, with 2,3-DPG and ATP being the most abundant, have the most influence on the oxygenation of Hb, thus affecting the oxyhemoglobin dissociation curve^(2,3)^.

## Phosphorus, diet and 2,3-diphosphoglycerate

Inorganic phosphorus (Pi) is an essential mineral that makes up 1 % of total body weight. Pi plays an important role in skeletal growth, mineral regulation and various cellular processes, including energy-transfer mechanisms, signal transduction, nucleotide metabolism and enzyme regulation^(4,5)^. In addition, Pi has been shown to modulate the oxygen release capacity of Hb by influencing the formation of 2,3-DPG^(6,7)^. Notably, Card *et al.* reported a positive correlation between plasma phosphate levels and erythrocyte ATP and 2,3-DPG^(8)^. This correlation suggests that 2,3-DPG levels are altered under conditions of hypo- or hyperphosphatemia.

In support, studies conducted on patients receiving continuous renal replacement therapy, which is known to have a net negative phosphate balance, showed a significant reduction in erythrocytes 2,3-DPG^(9)^. Moreover, hypophosphatemia associated with total parenteral nutrition causes a reduction in 2,3-DPG and increased Hb oxygen affinity^(10–13)^.

On the other hand, phosphorus loading was reported to increase plasma and erythrocytes 2,3-DPG levels and hence cause a right shift in the oxygen dissociation curve^(14–16)^. Hyperphosphatemia occurring in conditions such as uremia, post-transfusion analysis and after phosphorus infusion has also been found to increase 2,3-DPG concentration^(17,18)^. Under the condition of short-term high-altitude adaptation, the administration of low doses of phosphate supplementation for 4 days increased whole blood 2,3-DPG concentration, improving oxygen delivery and adaptation to high altitudes^(19)^. Similarly, a cross-sectional study on healthy subjects found that phosphate loading for 7 days increases both plasma and erythrocyte phosphate pools, resulting in a rise in erythrocyte 2,3-DPG concentration. Interestingly, a significant positive association was observed between erythrocyte 2,3-DPG and erythrocyte Pi levels, but not with plasma Pi levels^(14)^.

Furthermore, an *in vitro* study showed that 2,3-DPG production of erythrocytes incubated with inosine and pyruvate was augmented by the addition of phosphate at a level of 4 mM^(20)^. In line, administering a high phosphorus diet (1·5 %) in rats significantly increased the plasma 2,3-DPG concentration^(21)^. However, no information is available on the postprandial status of 2,3-DPG following the ingestion of meals with varied nutrient composition. In humans, a glucose load is associated with postprandial changes in many metabolites, including an increase in blood pyruvate^(22)^ and a decrease in plasma Pi^(23)^, which are known to have opposing effects on 2,3-DPG metabolism. Moreover, it is of interest to understand the impact of the phosphorus content of the meal on postprandial 2,3-DPG status. Postprandial reduction in 2,3-DPG would be expected to reduce oxygen availability to tissues and may reduce physical activity and cause sleepiness, which is a common observation following the ingestion of high-carbohydrate meals^(24)^.

Given their impact on 2,3-DPG levels and oxygen affinity, phosphorus supplements have been studied as a potential ergogenic aid for athletes. Under normal oxygen conditions, phosphorous supplementation enhanced aerobic exercise capacity by increasing erythrocyte 2,3-DPG and maximal oxygen consumption (VO_2max)_^(25,26)^. Contrarily, phosphate loading (1000 mg of tribasic sodium phosphate) for 6 days in competitive male runners significantly improved maximal and run performance despite no significant increase in erythrocyte 2,3-DPG levels being detected^(27)^. Similar results were observed in off-road cyclists, where both short-term (6 d) and long-term (21 d) sodium phosphate supplementation (50 mg/kg) significantly increased VO_2max_ and maximal aerobic power, with no significant changes in 2,3-DPG levels^(28)^. In support, 6 days of sodium phosphate supplementation (50 mg/kg) failed to influence serum phosphate levels and aerobic capacity in healthy, moderately trained men and women^(29)^. Similarly, the short-term impact of sodium phosphate supplements on time trial performance revealed no significant benefits^(30,31)^. However, the levels of 2,3-DPG were not determined in these supplementation studies^(29–31)^. A recent study investigating the effects of short-term phosphate loading on aerobic capacity under acute hypoxia in cyclists indicated that phosphate loading did not affect 2,3-DPG levels, Hb oxygen affinity, buffering capacity and myocardial efficiency^(32)^. The observed discrepancies between the studies may be attributed to the differences in the timing of 2,3-DPG assessment. Plasma phosphorus levels are known to increase following phosphate ingestion and return to baseline within a few hours, and 2,3-DPG may mimic such changes.

The impact of other micronutrients, such as thiamine, Zn, magnesium, vitamin E and cadmium, on 2,3-DPG levels has also been studied. Thiamine deficiency in rats was associated with reduced levels of erythrocytes 2,3-DPG^(33)^. Similarly, Zn deficiency significantly decreased 2,3-DPG per unit of packed red cells in male rats fed an egg white-based diet (containing < 1 mg/kg Zn) for 3 weeks^(34)^. On the other hand, a low dietary intake of Mg did not impact 2,3-DPG concentrations^(35)^. Incubating blood hemolysate with vitamin E for 4 h increased 2,3-DPG levels^(36)^. Furthermore, chronic oral cadmium administration in rats resulted in hypochromic anaemia and elevated levels of erythrocyte 2,3-DPG. This increase in 2,3-DPG may serve as a compensatory mechanism to enhance tissue oxygenation^(37)^.

Moreover, evidence that nucleosides (such as adenosine and inosine) increase 2,3-DPG levels in stored erythrocytes^(38)^ led Scopesi *et al.* to hypothesise that dietary nucleotide supplementation might have a similar effect *in vivo.* Accordingly, they investigated this hypothesis in both neonatal rats and preterm human neonates. In neonatal rats, nucleotide supplementation resulted in a significant increase in erythrocyte 2,3-DPG levels^(39)^. Conversely, in preterm human neonates, nucleotide-enriched formula did not produce a measurable effect on 2,3-DPG concentration^(40)^.

Furthermore, several dietary interventions have been shown to influence glycolysis, but their effects on the Rapoport-Luebering pathway in erythrocytes and subsequently 2,3-DPG have not been thoroughly studied. A study conducted by Xu *et al.* on Chinese participants found that short-term intensive fasting did not affect RBC 2,3-DPG levels, indicating no change in Hb’s oxygen-carrying capacity^(41)^. Conversely, caloric restriction resulted in a decrease in 2,3-DPG production among participants subjected to hypoxic conditions, which is contrary to the expected increase in response to hypoxia. This suggests that caloric restriction may override the expected hypoxia-induced increase in 2,3-DPG^(42)^.

Low-carbohydrate diets, such as the ketogenic diet, significantly reduce carbohydrate intake, resulting in a decrease in blood glucose levels and an increase in the production of ketone bodies^(43)^. These ketone bodies serve as an alternative source of energy for various cells except for erythrocytes. In fact, erythrocytes lack mitochondria, which are necessary for oxidative phosphorylation, the primary process by which ketone bodies are converted into energy^(44)^. Consequently, reduced glucose levels could potentially limit glycolytic flux in erythrocytes, although direct evidence is lacking. A study on the effects of a ketogenic diet in healthy females found no significant changes in complete blood count or erythrocyte morphology after 14 d. However, it did not assess erythrocyte 2,3-DPG levels, leaving the potential influence of the ketogenic diet on this critical glycolytic intermediate unexplored^(45)^.

The link between carbohydrate metabolism and 2,3-DPG levels is supported by evidence from an animal study, where administration of propane-1,2-diol led to elevated levels of blood glucose, lactate, pyruvate and an increased lactate-to-pyruvate ratio. These metabolic alterations were accompanied by a concomitant rise in erythrocyte 2,3-DPG levels, highlighting the influence of carbohydrate metabolism modulation on 2,3-DPG concentrations^(46)^.

Together, these findings highlight that dietary phosphorus is the most studied dietary factor, with clinically proven and strongly demonstrated effects on increasing erythrocyte 2,3-DPG levels and enhancing Hb oxygen release. In contrast, other micronutrients and dietary interventions remain poorly investigated, with most data limited to animal studies. Further research is needed to investigate the postprandial changes and the clinical relevance of modulating 2,3-DPG through diet.

## 2,3-Diphosphoglycerate metabolism

**Figure 1.**
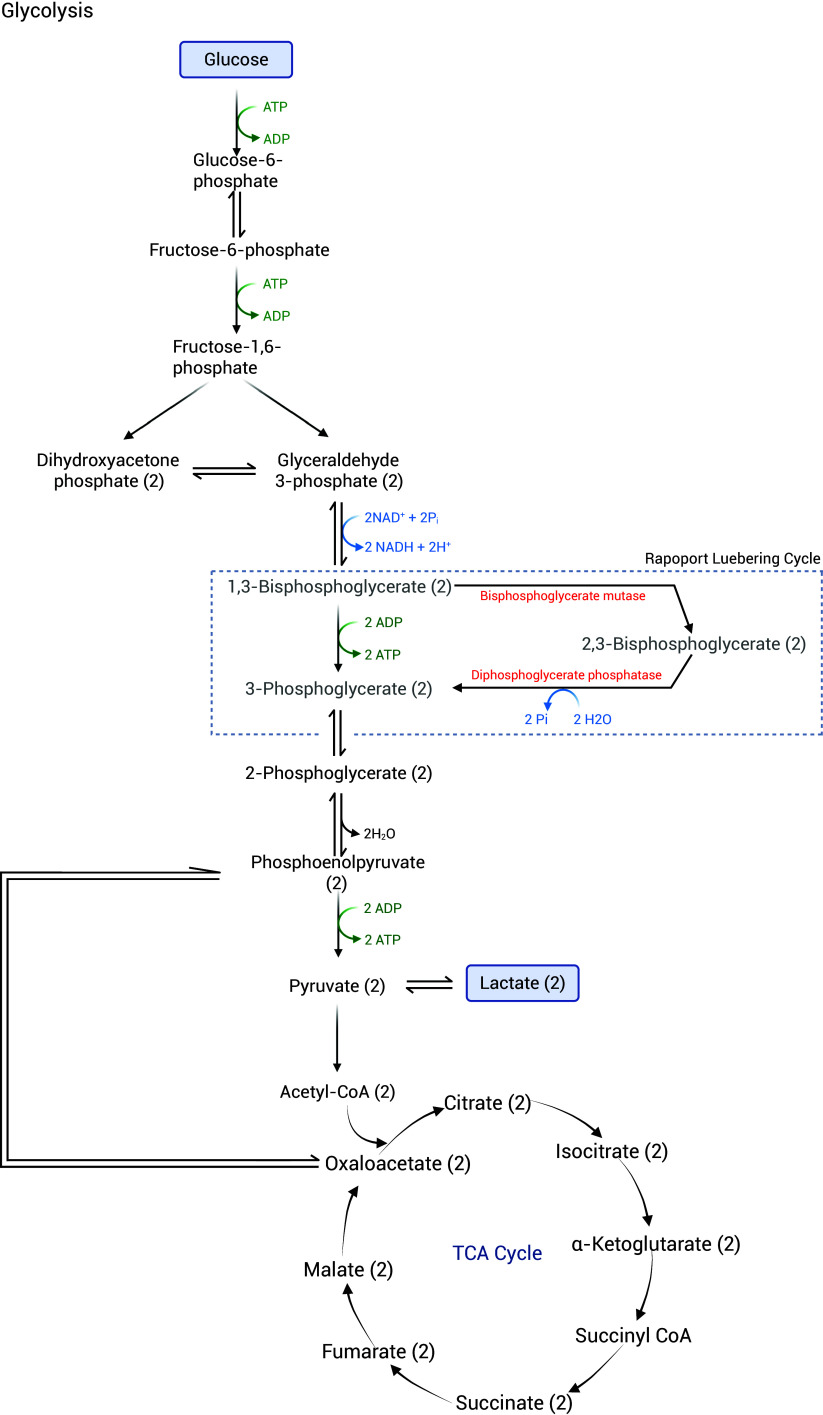
Outline of the glycolytic pathway showing the Rapoport Luebering Cycle. Created with BioRender.com.

In mature erythrocytes, 2,3-DPG is a metabolic intermediate in the Rapoport-Luebering pathway, a side shuttle to the anaerobic glycolytic pathway, also known as the Embden-Meyerhof pathway (Figure 1). It is synthesised from the isomerisation of 1,3-bisphosphoglycerate, as a phosphoryl group is transferred from C1 to C2 and catalysed by bisphosphoglycerate mutase. 2,3-DPG could be hydrolysed by phosphoglycerate phosphatase to 3-phosphoglycerate, releasing a Pi^(47)^. Around 20 % of the glycolytic pathway is fluxed via the Rapoport-Luebering shuttle, thus bypassing the direct conversion of 1,3-PBG to 3-phosphoglycerate, which would, under normal conditions, yield ATP, as mediated by phosphoglycerate kinase^(47,48)^.

Under normal physiological conditions, the production of 2,3-DPG is regulated by various mechanisms, including feedback inhibition, substrate availability and pH. Firstly, 2,3-DPG itself can inhibit the activity of 2,3-DPG mutase through a negative feedback mechanism^(49)^. Secondly, the level of 2,3-DPG can be affected by the availability of substrates such as phosphorus. Specifically, a decrease in 2,3-DPG levels is observed in cases of hypophosphatemia^(9)^, while an increase in 2,3-DPG levels is observed in cases of hyperphosphatemia^(8,14)^. Thirdly, pH is another factor affecting 2,3-DPG levels. In alkalotic conditions, the rate of glycolysis is increased, and the activity of 2,3-DPG phosphatase is diminished, leading to an increase in 2,3-DPG. In contrast, the levels of 2,3-DPG decrease during acidosis^(11,50)^. Finally, blockage in the glycolytic pathway can either increase or decrease 2,3-DPG levels depending on the location of the blockage and whether it is before or after the Rapoport-Luebering pathway. For instance, a deficiency in hexokinase blocks the glycolytic pathway proximal to 2,3-DPG production and subsequently reduces the level of 2,3-DPG. Inversely, a deficiency in pyruvate kinase blocks glycolysis below the shuttle, causing 2,3-DPG accumulation^(51)^. While thiamin deficiency, which is known to inhibit the activity of pyruvate dehydrogenase, was reported to reduce 2,3-DPG levels^(33)^. However, the ability of pyruvate to increase 2,3-DPG production is thought to be related to its capacity to increase NAD availability^(20)^.

Furthermore, numerous studies have shed light on the complex interactions among other physiological variables and the levels of 2,3-DPG. Nakashima *et al.* reported that the negative correlation between oxygen affinity of various mammalian Hb and body weight disappeared when 2,3-DPG was absent. This contrasts with the findings of Schmidt-Nielsen and Larimer^(52)^, who identified a negative correlation between these two factors in the presence of 2,3-DPG. This negative correlation was attributed to species-specific adaptations to optimal physiological conditions^(53)^. Additionally, a variation in 2,3-DPG levels between genders has been observed, with women displaying higher resting levels than men with comparable fitness levels. This difference may result from a compensatory mechanism in response to lower Hb levels in women^(54)^. Moreover, several hormones were also found to affect 2,3-DPG red cell concentration or oxygen affinity, including cortisol and aldosterone, androgen, growth hormone, thyroid hormone and erythropoietin. In 1968, Bauer and Rathschlag-Schaefer reported that the oxygen half-saturation pressure, a measure of oxygen affinity, was higher in aldosterone-treated and cortisol-treated groups compared with the control group^(55)^. Similarly, previous studies have shown a decreased oxygen affinity and a rise in erythrocyte 2,3-DPG levels with the administration of androgen^(56)^. Meanwhile, other studies demonstrated a synergistic effect of human growth hormone and thyroxine on the increase in erythrocyte 2,3-DPG concentration^(57)^. Versmold *et al.* demonstrated a significant correlation between thyroid hormone and red cell 2,3-DPG levels in infants, suggesting a role for thyroxine in 2,3-DPG synthesis, although the underlying mechanism remains unproven^(58)^. Finally, erythropoietin has also been shown to increase 2,3-DPG levels in erythrocytess^(59,60)^.

## Other allosteric effectors of Hb

In addition to 2,3-DPG, several endogenous and synthetic modulators have been shown to influence Hb oxygen affinity. Among these, sphingosine-1-phosphate has been reported to bind directly to Hb; however, its ability to reduce oxygen affinity appears to depend on the simultaneous presence of 2,3-DPG. This co-binding likely exerts a synergistic effect that stabilises the deoxygenated form of Hb, thereby promoting its low-affinity ‘tense’ state (T-state) conformation^(61)^. Indeed, Sun *et al.* reported that twenty-one healthy lowland participants ascending to 5260 meters had markedly elevated sphingosine-1-phosphate concentrations on the first day and continued to rise over the following 16 d. This upregulation was associated with the increased activity of sphingosine kinase 1, elevated Hb levels and enhanced oxygen-releasing capacity. Mechanistically, sphingosine-1-phosphate facilitates the binding of deoxygenated Hb to the erythrocyte membrane, promotes the translocation of glycolytic enzymes from the membrane to the cytosol, enhances glycolytic throughput and consequently increases intracellular 2,3-DPG, thereby promoting oxygen release^(62)^.

Pyridoxal 5’-phosphate, the active coenzyme form of vitamin B_6_, also functions as an allosteric modulator of Hb. It has been reported to reduce oxygen affinity and potentially compensate for diminished 2,3-DPG under certain metabolic conditions. However, when present in excess amounts within erythrocytes, pyridoxal 5’-phosphate appears to impair the synthesis of 2,3-DPG, likely due to the resulting decrease in intracellular pH and its binding with various intracellular enzymes^(63)^. Indeed, pyridoxal 5’-phosphate has been shown to inhibit key enzymes involved in both the glycolytic pathway and the pentose phosphate pathway, disrupting the metabolic pathways necessary for 2,3-DPG production^(64)^. Despite the potential physiological significance of these findings, there is a notable lack of recent studies exploring pyridoxal 5’-phosphate regulatory role in erythrocyte metabolism and its impact on 2,3-DPG synthesis.

Likewise, ATP has been recognised for its allosteric impact on Hb as demonstrated by Peng *et al.* Within physiological concentrations, ATP stabilises the reduced (Fe^2+^) form of Hb, promoting the deoxygenated (T) state conformation. However, at higher concentrations (4–7 mM), non-specific interactions may occur, leading to shifts in the oxidation potential. Unlike 2,3-DPG, which binds within the central cavity between the *β*-subunits, ATP interacts at different sites, resulting in a comparatively weaker allosteric effect on Hb^(65)^. Recent work by Parashar *et al.* proposes that Hb may catalyse ATP synthesis in erythrocytes under the murburn model. According to this model, when O_2_ levels are elevated and reducing equivalents such as reduced NADH are abundantly present, ample ATP is formed. The ATP binds to the heme pocket of Hb and prevents the premature oxygen dissociation and enhances oxygen retention during transport^(66)^. Interestingly, in a crossover study simulating 3500 m altitude hypoxia, Woyke *et al.* assessed the oxygen dissociation curve, erythrocyte 2,3-DPG and ATP concentrations. They observed a significant increase in 2,3-DPG that elevated P_50_ and offset respiratory alkalosis, thereby maintaining effective oxygen delivery to tissues. In contrast, ATP concentrations remained largely unchanged and thus did not significantly influence Hb oxygen affinity during short-term hypoxic exposure^(67)^.

The role of 2,3-DPG in O_2_ unloading has led to the development of synthetic allosteric effectors aimed at modulating Hb oxygen affinity in clinical contexts, especially for the treatment of ischaemia-related diseases such as sickle cell disease^(68)^. Compounds such as inositol hexaphosphate, bezafibrate and efaproxiral have been shown to reduce Hb’s oxygen affinity by shifting the allosteric equilibrium towards the T-state, thereby enhancing oxygen delivery^(68–70)^. In contrast, various natural and synthetic compounds have been reported to shift Hb’s allosteric equilibrium towards a high-affinity state and have also been investigated as potential therapeutic agents for the treatment of sickle cell disease. Aromatic aldehydes, including vanillin and 5-hydoxymethyl-2-furfural, have been shown to increase oxygen affinity by interacting with Hb^(68)^. Specifically, they shift the Hb equilibrium toward the ‘relaxed’ state (R-state) leading to a leftward shift in the oxygen dissociation curve^(71,72)^. However, vanillin requires relatively high concentrations to exert significant effects on Hb oxygen affinity and is limited by poor bioavailability. In contrast, 5-hydoxymethyl-2-furfural has shown greater potency, prompting the development of more effective derivatives^(68)^.

## 2,3-Diphosphoglycerate assessment

The stability of a compound in its physiological milieu dictates its accurate measurement. The concentration of 2,3-DPG was reported to be influenced by various factors, including storage lesion, which refers to the storage-induced alterations or damage in the erythrocytes. In fact, delayed refrigeration of blood after collection leads to lactate accumulation, resulting in a drop in blood pH. This decrease in pH activates bisphosphoglycerate phosphatase, causing a rapid depletion of 2,3-DPG levels^(73)^. Therefore, addressing this concern is of importance in blood transfusion since transient depletion of 2,3-DPG will increase the affinity of oxygen to Hb and reduce oxygen delivery to tissues, consequently impacting posttransfusion recovery. This is clinically significant, particularly in severely ill patients who have undergone massive transfusions and infants under four months old, as they have a reduced ability to replenish their 2,3-DPG levels and compensate for hypoxemia and its effects^(73,74)^. In response, various procedures were implemented to improve the preservation of 2,3-DPG in blood samples. These included adding additives, such as guanosine, inosine, phosphate, pyruvate, sodium chloride, etc., to modify the pH or affect the metabolism of erythrocytes^(73)^. In brief, 2,3-DPG’s degradation rate depends on the preservation solution in which the erythrocytes are stored as well as the handling procedures prior to storage at +4℃. Thus, immediate cooling of whole blood after collection can significantly prevent the rapid fall in 2,3-DPG levels^(75)^.

On the other hand, several methods for the assessment of the erythrocyte concentration of 2,3-DPG have been described in the literature and are divided into two main categories: chromatographic and enzymatic. Barlett *et al.*, in 1959, first described the chromatographic technique in which an ion exchange column isolates phosphate compounds, and then 2,3-DPG is hydrolysed into glyceric acid and phosphorous to be quantified. This method was later modified by other researchers to also isolate and remove nucleotides, which were reported to be the second most important phosphorous-containing compounds^(76–79)^. On the other hand, the enzymatic methods were based on the catalytic effect of 2,3-DPG on phosphoglycerate mutase and that phosphoglycerate mutase exhibits 2,3-DPG phosphatase activity^(80)^. A study comparing five methods for the determination of 2,3-DPG in blood, which included a colorimetric method, two enzymatic endpoint methods and two enzymatic rate-dependent methods, concluded that there were no statistically significant differences between them^(81)^. Commercial kits for the quantification of 2,3-DPG based on the enzymatic method from reputable companies have been discontinued. Although other commercial ELISA kits are still available, their accuracy is questioned, as numerous customer reviews indicate that sample analysis often yields unexpectedly low 2,3-DPG values when higher levels are anticipated.

## 2,3-Diphosphoglycerate functions

**Figure 2.**
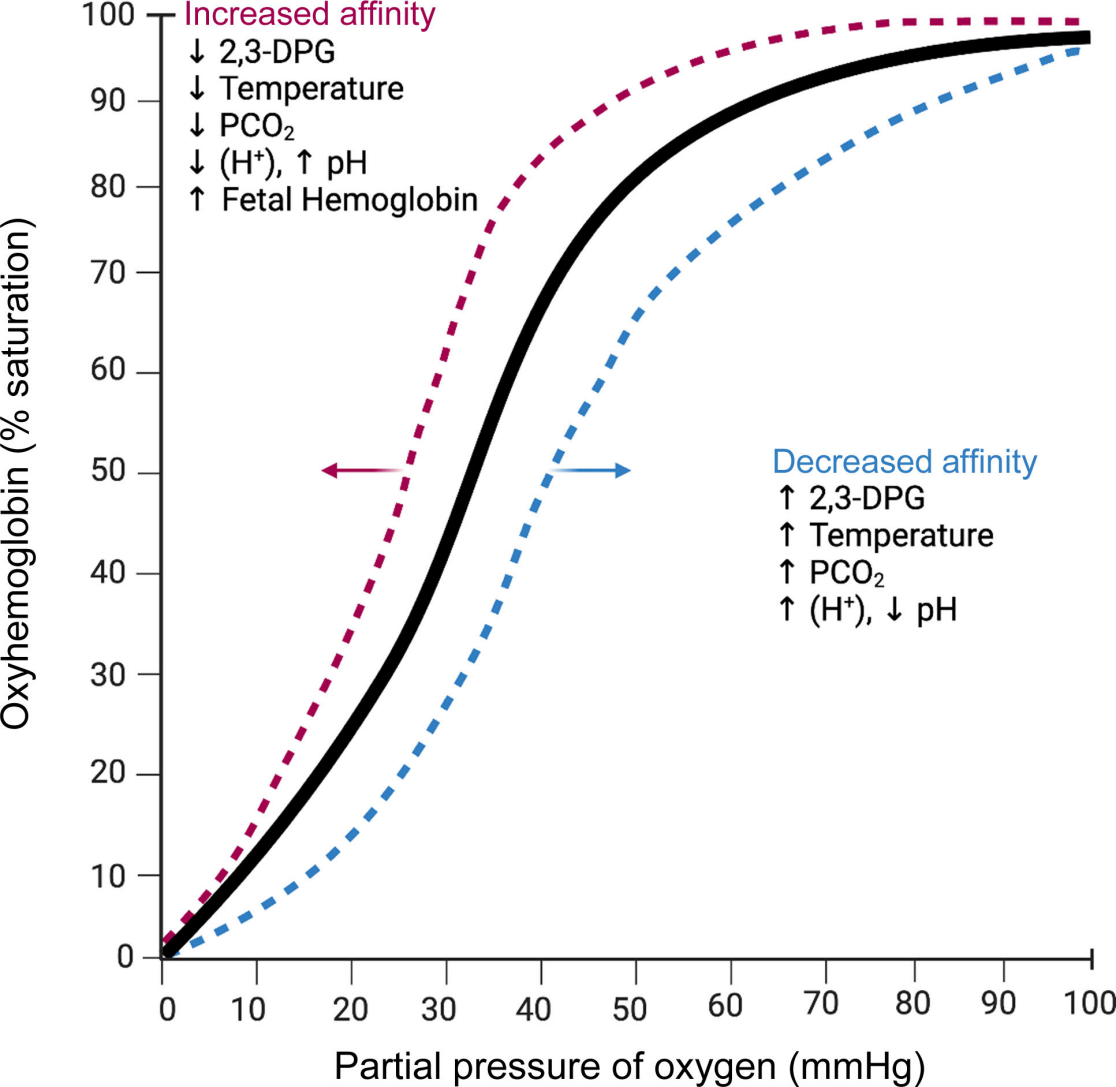
Factors affecting the oxygen–hemoglobin dissociation curve. Modified from Darlow *et al.* ^(82)^. Created with BioRender.com.

2,3-DPG has the capacity to exert a potent effect on the sigmoid-shaped oxyhemoglobin dissociation curve. This curve is achieved by plotting the percent saturation of Hb against the oxygen tension, pO_2_, demonstrating the affinity of Hb for oxygen. The position of the curve in relation to the partial pressure or oxygen tension influences oxygen delivery to the tissues. P50, which is the oxygen tension at which 50 % of the Hb is saturated under fixed conditions, plays an important role as a reference point when assessing the effect of various factors on the curve^(80)^. As the 2,3-DPG level in the erythrocyte increases, the oxygen dissociation curve shifts to the right, thus decreasing the oxygen affinity of Hb. As its concentration decreases, the oxygen dissociation curve shifts to the left, and consequently, the affinity of Hb for oxygen increases, thus binding tightly to the oxygen molecule. However, it is important to note that several other factors may influence the oxygen dissociation curve, including pH, temperature, partial pressure of carbon dioxide, fetal Hb, etc. (Figure 2)^(83)^.

**Figure 3.**
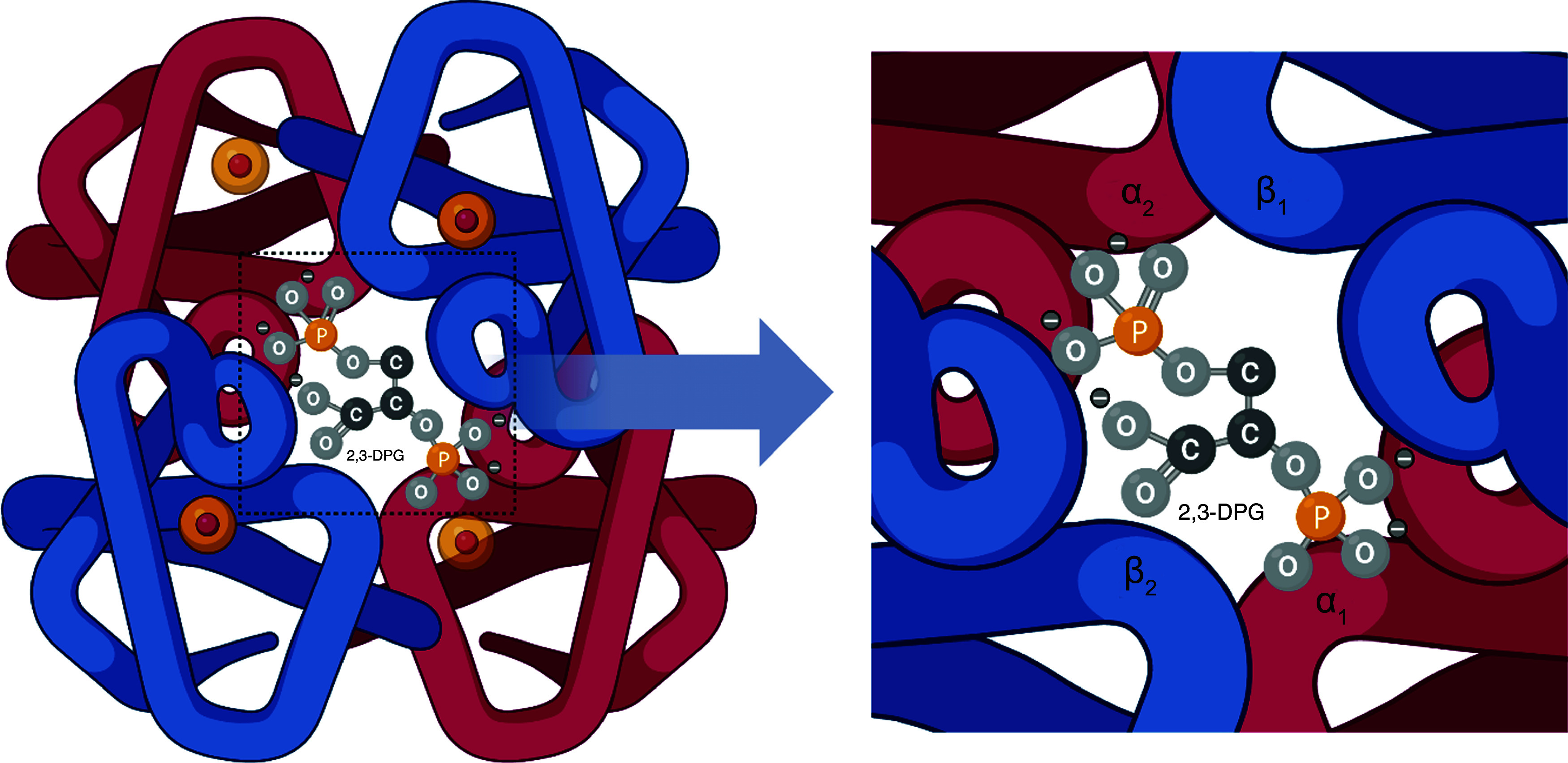
The binding of 2,3-diphosphoglycerate to deoxyhemoglobin. Modified from Mathews *et. al*.^(85)^. Created with BioRender.com.

Using X-ray crystallography, Arthur Arnone reported that 2,3-DPG has two effects on human deoxyhemoglobin: it stabilises the Hb structure in the deoxyhemoglobin form and shortens the distance between the two *β*-subunits^(84)^. However, several studies reported that 2,3-DPG did not exert the same effect on an oxyhemoglobin molecule, as it is postulated that the site of binding in the deoxy-form is absent in the oxy-form (Figure 3)^(84,86)^. Deoxygenated Hb is found in the T-state, and the binding of one oxygen molecule to it would result in a transition to the R-state, which enhances the binding of oxygen to the other subunits of the deoxygenated Hb. 2,3-DPG, being negatively charged, fits between the *β*-subunits, inhibiting their movement and stabilising the deoxy form. The oxygenated form lacks this binding site, as the *β*-subunits are close together, making the cleft between them too small for 2,3-DPG to enter^(83)^. Another mechanism by which 2,3-DPG reduces the oxygen affinity of Hb within erythrocytes could involve changes in the hydrogen concentration, leading to decreased intracellular pH levels^(15)^. In brief, 2,3-DPG function is related to its capacity to stabilise deoxyhemoglobin T-state and lower intracellular pH.

## 2,3-diphosphoglycerate, hypoxia and altitude

Hypoxia, characterised by low tissue O_2_ levels, can result from a variety of pathological and physiological conditions. Pathologically, it is commonly associated with diseases that lead to low blood supply or reduced O_2_ content in the blood, such as cardiovascular^(87,88)^, respiratory^(89,90)^ and haemolytic diseases^(91,92)^. In contrast, physiological hypoxia may manifest in healthy individuals residing at high altitudes^(93,94)^. The state of hypoxia, regardless of its pathological or physiological origin, elicits rapid adaptive responses from the body to cope with its reduced oxygen availability. One such response involves the reduction of Hb-O_2_ affinity by modifying the levels of 2,3-DPG. It has been shown that 2,3-DPG concentrations increase in patients with cognitive heart failure, myocardial infarction and peripheral vascular disease to compensate for the reduced oxygen supply^(88,95–97)^.

In healthy individuals, the concentration of 2,3-DPG increases as a natural response to altitude. At sea level, the concentration of 2,3-DPG is around 90 ± 11 µg phosphorus/ml of blood. Upon moving to high altitude, this concentration rises to about 142 ± 8 µg phosphorus/ml of blood within 24 h. When returning to sea level, the concentration reverts back to baseline and reaches around 87 ± 13 µg/ml. This highlights the physiological role of 2,3-DPG in the body’s adaptive mechanisms^(93)^. Moreover, altitude training in athletes has been reported to increase erythropoietin production, which is known to increase 2,3-DPG^(59,60)^. The extent of the rise in 2,3-DPG levels was shown to depend on both the level of altitude and the duration of exposure, with erythropoietin levels peaking at 24 h after exposure to high altitude^(98)^.

**Figure 4.**
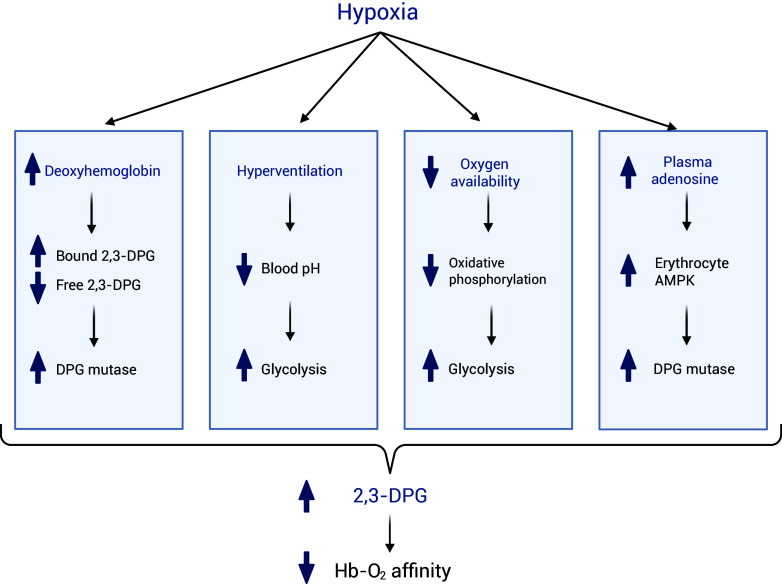
Summary of the mechanisms that lead to an increase in 2,3-DPG production under hypoxic conditions. Created with BioRender.com.

Several explanations were proposed for the mechanisms behind the increase in 2,3-DPG in response to hypoxia (Figure 4). In hypoxic conditions, the increase in deoxyhemoglobin leads to more 2,3-DPG binding, thereby reducing the levels of free 2,3-DPG. This decrease in free 2,3-DPG levels reactivates 2,3-DPG mutase through a feedback mechanism, ultimately increasing 2,3-DPG synthesis^(99)^. Additionally, hypoxia induces hyperventilation, resulting in respiratory alkalosis and an elevated blood pH^(100)^. This increase in pH stimulates glycolysis, contributing to an increase in the concentration of 2,3-DPG^(99)^. Furthermore, in cases where tissue oxygen demand surpasses its supply, cells must rely on anaerobic metabolism to produce sufficient ATP for their energy requirements^(101)^. This shift in metabolism results in an increase in the rate of glycolysis, which may lead to an elevation in the levels of 2,3-DPG. Moreover, Liu *et al.* conducted studies on humans at high altitude and on mice to investigate the mechanism of hypoxia adaptation. Their findings showed that the increased plasma adenosine, that occurs under hypoxic conditions, activates erythrocytes’ AMP-activated protein kinase (AMPK). In turn, AMPK activates DPG mutase, increasing 2,3-DPG production and improving O_2_ release^(102)^.

## 2,3-DPG and physiological conditions

The process of transporting oxygen from the lungs to the tissues involves a series of events. It all begins with ventilation, which is the inflow and outflow of air into the alveoli. Following this, O_2_ diffuses from the lungs into the RBCs and binds to Hb. When oxygenated RBCs travel through the bloodstream, the O_2_ diffuses from Hb into the mitochondria within the cells^(103)^. The transport of O_2_ via Hb is a crucial step in this process, which can be enhanced by either increasing Hb concentration per unit volume of blood or by altering Hb’s affinity to O_2_. Hb concentration rises slowly, and the increase usually occurs in response to long-term adaptation to hypoxia or training^(98,104)^. In contrast, the control of Hb-O_2_ affinity occurs quickly as RBCs pass through the capillaries and encounter changes in temperature, pH and/or CO_2_^(105)^. Hence, in conditions that demand prompt physiological responses from the body, the affinity of O_2_ to Hb is altered. This can be chiefly achieved through the use of endogenous allosteric modulators that affect Hb-O_2_ affinity, particularly 2,3-DPG^(99)^. However, it is reasonable to postulate that under conditions of high energy demand (increased oxygen requirement) (e.g., growth, pregnancy, physical activity), an adaptive mechanism would be required to accommodate the extra need for oxygen.

### Infancy and childhood

**Figure 5.**
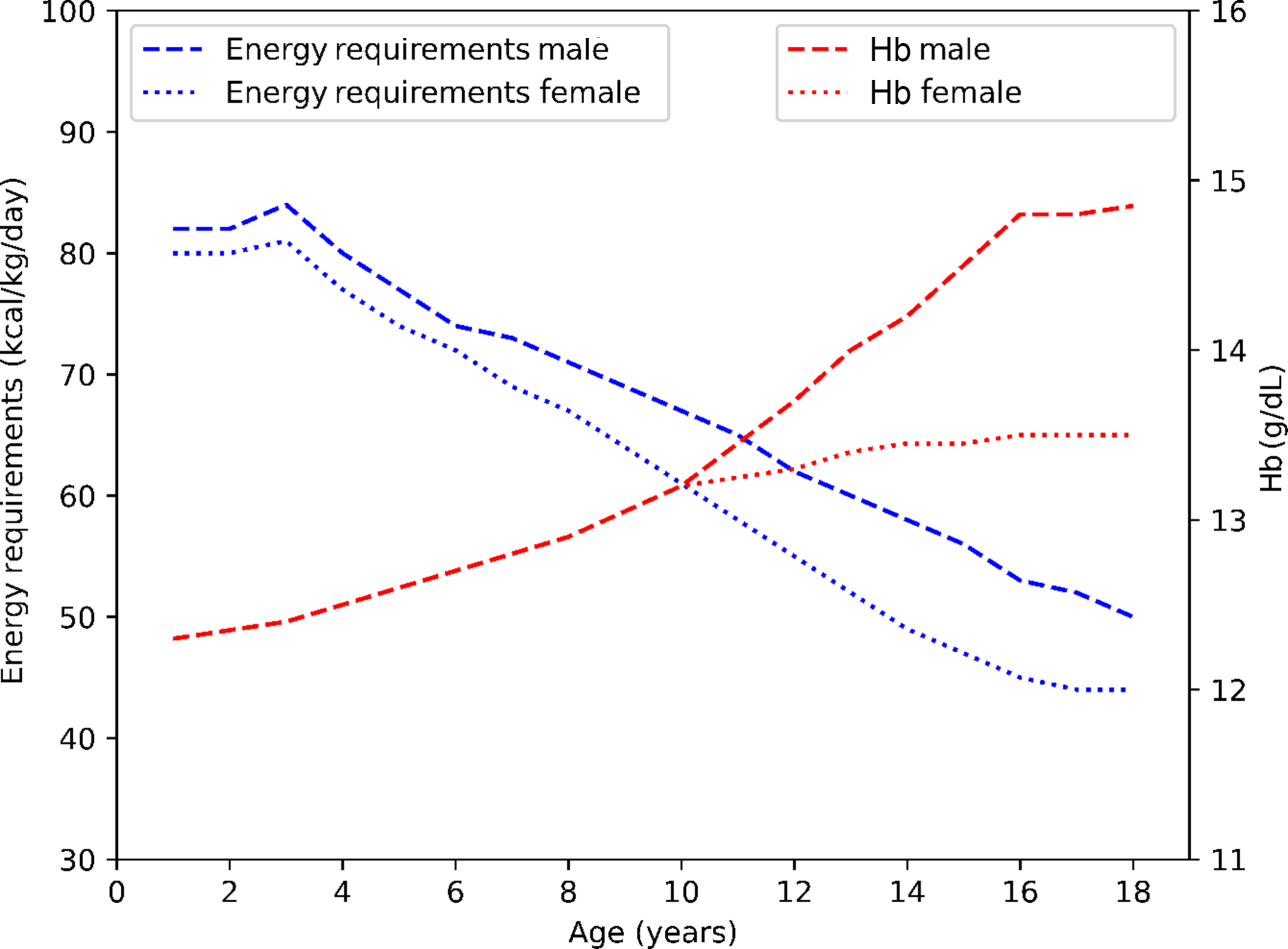
Energy Requirements (2004 FAO/WHO/UNU)^(110)^ and median Hb levels by age and sex. Modified from Butte, N. F.^(111)^ and Yip *et al.* ^(112)^.

Total energy expenditure expressed per Kg body weight is known to be highest in early life (∼80 kcal/kg) and decreases with age (∼ 40 kcal/kg in adults)^(80,81)^. In contrast, blood Hb increases with age, with Hb concentration exhibiting its lowest values in early life^(106–109)^. This inverse relation between energy expenditure (kcal/kg body weight) and Hb necessitates the existence of a mechanism to cater to the need for oxygen by tissues (Figure 5). On the other hand, the pattern of changes in 2,3-DPG with age seems to mimic that of energy expenditure and thus may be implicated in the process of oxygen delivery. In fact, the level of 2,3-DPG was reported to be highest in infancy and decreases with age^(113,114)^. Therefore, it is reasonable to propose that this relationship exists to facilitate oxygen supply and conquer the low levels of Hb observed during infancy and childhood. In support, children are known to have higher serum and red cell organic phosphates as compared to adults, resulting in age-specific physiological hyperphosphatemia. This, in turn, is proposed to increase 2,3-DPG concentration and reduce oxygen affinity, which may explain the physiological anaemia observed in childhood^(8)^. Unfortunately, the existing data on variations in erythrocyte 2,3-DPG levels across different age groups in the general population is limited by a broad 10-year age range, which may obscure more precise age-related changes. Though, 2,3-DPG level of a small number of newborns (*n* 12) was reported to increase during the first two weeks of life, and this was paralleled by the changes in thyroxine^(58)^. Additionally, a transition from fetal H to adult Hb occurs during infancy^(115)^. This transition can potentially impact the levels of 2,3-DPG, as these two forms of Hb have different affinities to oxygen^(116,117)^. In summary, it is recommended that future research use narrower age intervals to gain a more precise understanding of 2,3-DPG level variations across different age groups.

### Pregnancy

Pregnancy, characterised by increased energy demands to support maternal weight gain, placental development and fetal growth, is also expected to be associated with alterations in 2,3-DPG levels. 2,3-DPG level was reported to increase significantly from the first to the third trimester (16·1–17·0 µmol/g Hb, *P* < 0·01) and then decrease after delivery^(118)^. In line with this, a longitudinal study showed that 2,3-DPG levels were higher in pregnant than in non-pregnant women. This increase in 2,3-DPG levels may be related to respiratory alkalosis, hormone effects and relative anaemia during pregnancy^(119)^. A comparison of 2,3-DPG levels in anaemic and non-anaemic pregnant women revealed an increase in both groups between the 16th and 42nd weeks of pregnancy. However, anaemic pregnant women had higher levels of 2,3-DPG at each stage compared with normal pregnant women^(120)^. In brief, the 2,3-DPG levels increase during pregnancy and are influenced by both gestational age and anaemic status. This increase lowers the oxygen affinity to maternal Hb, thereby facilitating the efficient transport of oxygen to the fetus across the placenta.

### Physical activity

Studies on the effect of exercise type, duration and intensity on the levels of 2,3-DPG yielded inconsistent and contradictory findings. In their study, Ricci *et al.* collected blood samples from runners immediately after exercise, and they found that the post-exercise 2,3-DPG levels did not show a significant increase compared with pre-exercise levels. However, 2,3-DPG levels were notably higher in the runners compared with untrained controls^(121)^. Similar results were reported by Bonner *et al.* in untrained women who were subjected to walking treadmill tests^(122)^. However, several studies have highlighted that the timing of blood collection significantly influences 2,3-DPG measurements, with blood samples collected immediately after exercise showing notably lower values^(123,124)^. Alternatively, Lijnen *et al.* assessed variations in blood 2,3-DPG levels in marathon runners at different time points, including baseline, immediately post-race, 12 and 36 h post-race. The authors reported a significant increase in 2,3-DPG levels immediately after the marathon, with sustained elevation up to 12 h post-race. Subsequently, levels returned to pre-race values 36 h after the marathon^(125)^. In line, Meen *et al.* showed that heavy exercise, whether short or long lasting, leads to a definite increase in 2,3-DPG levels that lasts for several hours. The authors noted that the rate of increase in 2,3-DPG levels varies depending on the type of exercise^(126)^. These contradictory results emphasise the necessity to collect blood samples at multiple time points to evaluate the influence of exercise on 2,3-DPG levels accurately.

It is noteworthy that existing literature has predominantly focused on evaluating the impact of physical exercise on 2,3-DPG levels under normoxic conditions. A recent study conducted by Ploszczyca *et al.* aimed to assess the effect of prolonged intense exercise under normoxic and hypoxic conditions on 2,3-DPG levels in cyclists. The authors concluded that intense exercise in hypoxic conditions leads to a decrease in 2,3-DPG concentration as compared with normoxia, and this is primarily due to exercise-induced acidosis^(127)^. However, this study’s participants were placed directly into hypoxic conditions without an adaptation period. Previous studies showed that 2,3-DPG increases within 24 h in response to high altitude/hypoxia^(93)^. Therefore, the lack of an adaptation period may have influenced the findings.

Moreover, exercise intensity is another factor that can influence the levels of 2,3-DPG. For instance, Hsieh *et al.* showed that low-intensity aerobic exercise (35 % VO_2max_) does not have a significant effect on 2,3-DPG levels, whereas moderately high-intensity exercise (75 % VO_2max_) leads to an increase in its concentration^(128)^. Most studies to date have investigated the impact of submaximal workloads on 2,3-DPG, yet the effects of anaerobic work, particularly resistance training, have received less attention.

## 2,3-Diphosphoglycerate and various pathological conditions

The body’s functions are regulated by an interrelated networking of biochemical processes. While 2,3-DPG plays a crucial role in physiological activities through the control of oxygen affinity, it has been found to be implicated in several diseases and disorders. A significant association between this molecule and various diseases can be attributed to its involvement in hypoxic conditions. Therefore, in most cases, the variation of 2,3-DPG levels is often a consequence rather than a cause.

Anaemia, especially iron deficiency anaemia, is also considered a substantial contributor to hypoxia. Iron deficiency anaemia is characterised by a drop in the blood levels of Hb, resulting in a reduced oxygen-carrying capacity of the blood (hypoxia)^(129)^. Consequently, an elevation in 2,3-DPG levels is expected during anaemic states, reflecting an analogous physiological response to that observed in other hypoxic conditions. Studies conducted on iron deficiency anaemia in both adults and children have provided supporting evidence for the inverse association between 2,3-DPG and Hb levels^(130–132)^. However, this association may not be absolute, as other factors such as the pulmonary status, tissue oxygen demands, intracellular environment and potentially even the age of cells could also exert influence on the levels of 2,3-DPG^(132)^. Interestingly, the degree to which the concentration of 2,3-DPG increases can differ among various types of anaemia, even when the Hb concentration is the same^(133)^. A smaller increase in 2,3-DPG was observed in aplastic anaemia as compared with other forms of anaemia^(134)^. Furthermore, it has been suggested that erythrocyte mass may serve as a more sensitive indicator for predicting 2,3-DPG concentration compared with Hb. This is attributed to the correlation between 2,3-DPG levels and the deficiency in erythrocyte volume^(135)^. Therefore, the significance of 2,3-DPG in anaemia has been widely discussed, but its molecular mechanism is still unclear and needs further elucidation.

An increase in 2,3-DPG has been reported in subjects with pulmonary insufficiency and congestive heart failure, conditions in which hypoxia predominates; hence, the need to lower oxygen affinity. In chronic pulmonary diseases, 2,3-DPG is stimulated by reduced oxygen saturation; however, in acute cases, the alteration of the pH drives the increase or decrease of 2,3-DPG. Alkalosis stimulates the glycolytic pathway and thus elevates 2,3-DPG, while acidosis inhibits the pathway and results in a decreased concentration^(136)^.

The involvement of 2,3-DPG is not limited to blood, lung and heart diseases. Individuals diagnosed with Parkinson’s disease exhibit an increase in 2,3-DPG concentration, even after adjusting for Hb levels. This may have been attributed to respiratory difficulties related to postural abnormalities and rigidity, leading to hypoventilation and hypoxia. 2,3-DPG is also thought to be involved in the cholinergic and dopaminergic systems, where an imbalance is noted in Parkinson’s disease^(137)^. Another study has postulated that the increase in 2,3-DPG is a metabolic compensatory mechanism to the high-affinity Hb and the inefficiency of releasing oxygen in patients with Parkinson’s disease^(138)^.

On the other hand, both increased oxidative stress, as indicated by the reduction of erythrocyte glutathione peroxidase, and decreased 2,3-DPG concentrations were hypothesised to play a role in the pathophysiology of dementia-related conditions and contribute to cognitive impairment in Alzheimer’s disease^(139)^. However, the similarity in erythrocyte 2,3-DPG levels in patients with Alzheimer’s disease with or without dementia argues against this hypothesis and implies that oxygen-regulatory pathways do not participate in brain hypoxia^(140)^.

Aside from hypoxia, 2,3-DPG was proposed to have an anti-platelet aggregation function. Subjects with hypochromic anaemia are known to have decreased levels of platelet aggregation, while those with polycythemia have a common incidence of thrombosis. Like in iron deficiency anaemia, subjects with hypochromic anaemia have higher levels of 2,3-DPG^(141)^, while the latter decreases in polycythemia^(142)^. The difference in platelet aggregation between hypochromic and polycythemia was attributed to 2,3-DPG^(143)^.

Moreover, the observed drop in 2,3-DPG among patients with kidney failure may be due to metabolic acidosis^(144)^, a common condition in chronic kidney diseases^(145)^. However, a noticeable elevation of erythrocyte concentrations of 2,3-DPG was reported among non-acidotic anemic patients who underwent haemodialysis, another coping mechanism to counter anaemia of renal disease and a rise of around 50 % was seen in cases of end-stage renal disease^(146)^.

The fact that 2,3-DPG is a bioproduct in the glycolytic pathway may implicate it in glucose metabolism and diabetes. In a clinical study, compared with healthy participants, diabetic subjects had a significant increase in 2,3-DPG irrespective of the type and severity of the disease^(147)^. This increase was believed to reverse the shift to the left of the oxygen dissociation curve caused by the glycation of Hb^(148)^ and may also be also related to hypoxia of diabetes^(149)^. Nonetheless, these findings have not been supported by others^(150,151)^, and some associations have been made between the increase of 2,3-DPG only in cases of diabetes with vascular complications^(97)^. Interestingly, the treatment of diabetes seemed to affect neuropathy when 2,3-DPG levels are low. Insulin-treated streptozotocin rats had higher degeneration levels of their nerves when 2,3-DPG concentrations were lower, while untreated rats showed no damage^(152)^. In addition, diabetic ketoacidosis is a known complication of diabetes, especially type 1. It is characterised by a sharp increase in hydrogen ions that affect blood pH^(153)^, which hinders erythrocyte glycolysis, mainly through the inhibition of phosphofructokinase, which decreases 2,3-DPG. Moreover, in a study using a nonobese prediabetic rat model, the increased phosphorus content in the diet, which is known to increase 2,3-DPG^(21)^, was associated with a reduction in hypoxia-inducible factor-1*α* in the perivascular adipose tissue^(154)^. Although 2,3-DPG was not measured in the previous study, Guoji *et al.* have demonstrated a suppressive effect of the bisphosphoglycerate mutase/2,3-DPG pathway on hypoxia-inducible factor-1*α* in hypoxic astrocytes^(155)^. Hence, it is postulated that 2,3-DGP mitigates perivascular adipose inflammation and its cardiovascular consequences in early metabolic impairment.

Obesity is a condition associated with numerous metabolic disturbances, among which is the increase in 2,3-DPG^(150)^. This may be attributed to the respiratory distress people with obesity suffer from^(156)^. On the other hand, low 2,3-DPG levels may be involved in the pathogenesis of obesity. Decreased 2,3-DPG causes a decrease in oxygen availability, which can lead to diminished physical activity capacity and, consequently, lower energy expenditure^(157)^.

In summary, the metabolism of 2,3-DPG, a by-product of glycolysis, is influenced by several factors, including diet, physiological and pathological conditions (Table 1). The influence of the diet composition on 2,3-DPG levels is far from clear, and information on its postprandial metabolism is lacking. In fact, the diet seems to acutely impact the levels of 2,3-DPG, unlike that of altitude, which requires about 36 h. Discrepancies between studies seem to relate to the time and method of measurement. Therefore, increased 2,3-DPG levels may partially mitigate conditions of low oxygen availability, including anaemia and hypoxic conditions. However, the precise implications of this interaction during infancy and childhood remain unclear. Additionally, data do support a role for 2,3-DPG in platelet aggregation and neurodegenerative diseases, and further studies are needed to better understand these effects.

**Table 1.** Physiological and pathological factors influencing 2,3-DPG, Hb and HIF-1*α*

Condition/state	2,3-DPG levels and other parameters such as [Hb] and [HIF-1*α*]	References
Physiological conditions		
Age	↓ [2,3-DPG] with age ↑ [Hb] with age	^(106,114)^
Hypoxia/high altitude	↑ [2,3-DPG] ↑ [HIF-1*α*]	^(93,98,158,159)^
Pregnancy	↑ [2,3-DPG] from the first to the third trimester and ↓ after delivery ↓ [Hb] during the first and second trimesters and then ↑ during the third	^(119,160)^
Physical activity	Depending on exercise type, duration and intensity	^(105,121–126,128,159)^
Phosphorus levels	↑ [2,3-DPG] with phosphorus supplementation and hyperphosphatemia ↓ with hypophosphatemia ↓ [HIF-1*α*] with increased phosphorus	^(11–16,18)^
Pathological conditions		
Fe deficiency anaemia	↑ [2,3-DPG] ↓ [Hb] ↑ [HIF-1*α*]	^(129–132, 161,162)^
Chronic pulmonary and CVD	↑ [2,3-DPG]	^(136)^
Alzheimer	↓ [2,3-DPG]	^(139)^
Parkinson’s disease	↑ [2,3-DPG]	^(137,138)^
Diabetes	↑ [2,3-DPG]	^(147)^
Obesity	↑ [2,3-DPG]	^(151)^
Kidney failure	↓ [2,3-DPG]	^(144)^

HIF-1*α*, hypoxia-inducible factor-1α; 2,3-DPG, 2,3-diphosphoglycerate.
